# Supplementary material for: Transcriptome-Wide Analysis of microRNA–mRNA Correlations in Tissue Identifies microRNA Targeting Determinants
Source: Noncoding RNA. 2023 Feb 13;9(1):15. doi: 10.3390/ncrna9010015 (PMC9958706; doi:10.3390/ncrna9010015)
Supplement: Supplementary file 1 [file ncrna-09-00015-s001.zip › Supplementary material Figures.pdf]

FIGURES

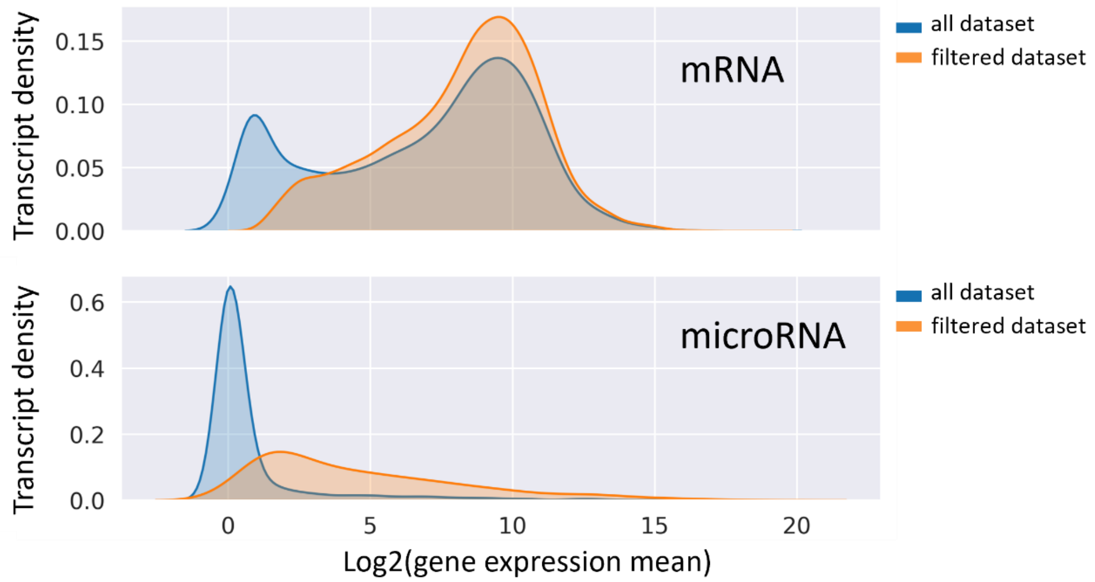

Figure S1. Density distribution of microRNA and mRNA expression on the TCGA-PRAD dataset before and after filtering for detection in 80% of the samples.

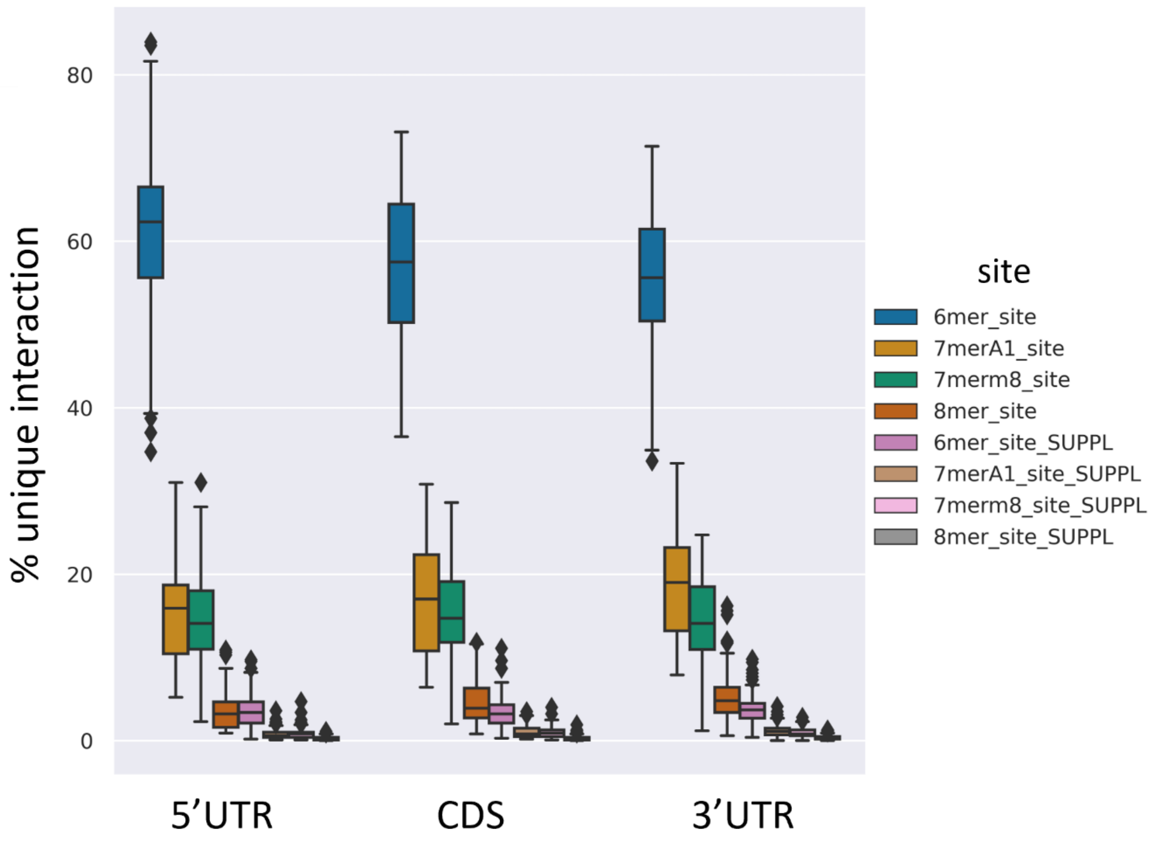

Figure S2. *Percentage of unique interactions per microRNA site types by the transcript regions*, as indicated in the side-colored labels and the x-axis labels. Data calculated for the 143 filtered in microRNAs.

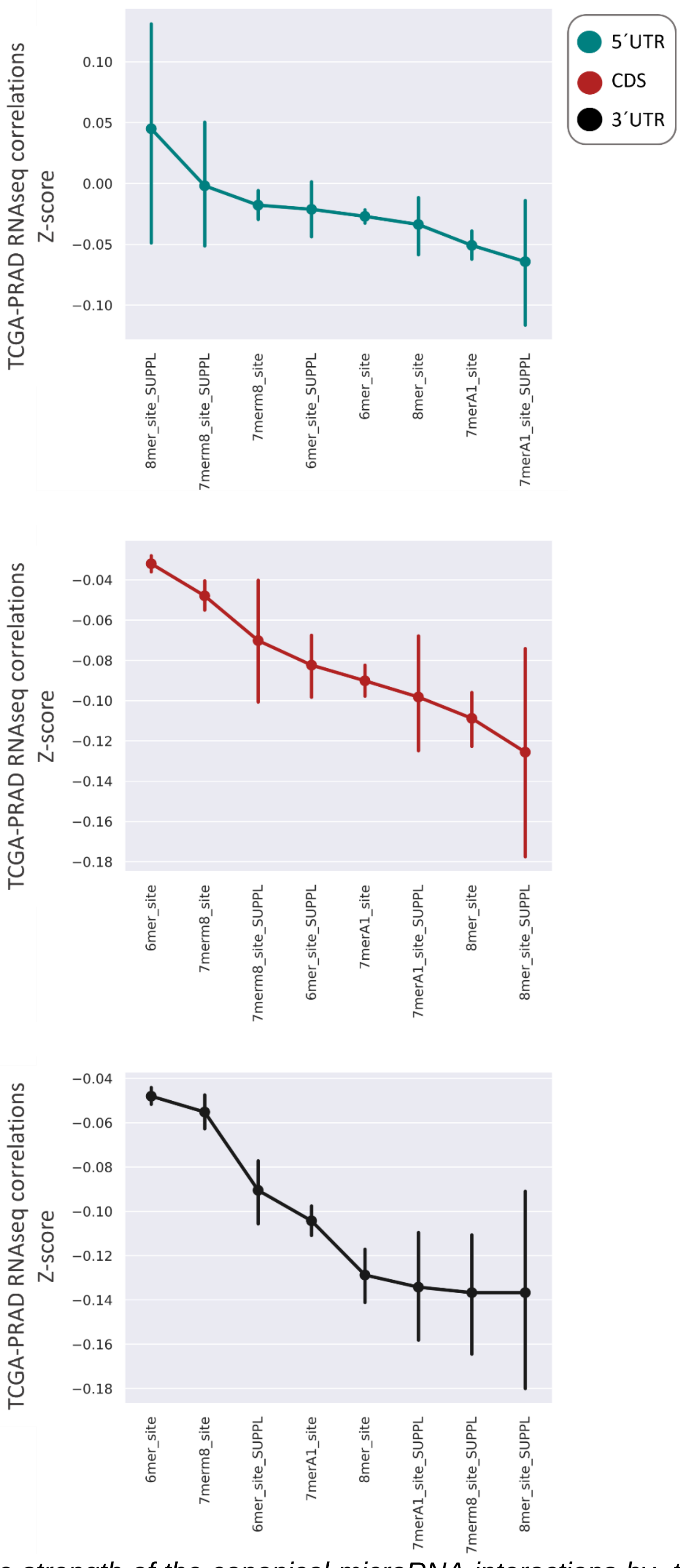

Figure S3. *Repressive strength of the canonical microRNA interactions by transcript region.* Data from Figure 2 was plotted by repressive strength order. The average Z-transformed correlations between the 143 conserved microRNAs and their predicted target mRNA are represented for the coding regions (CDS) and the 5´ and 3´ untranslated regions (UTR). Vertical bars represent the confidence interval (95%).

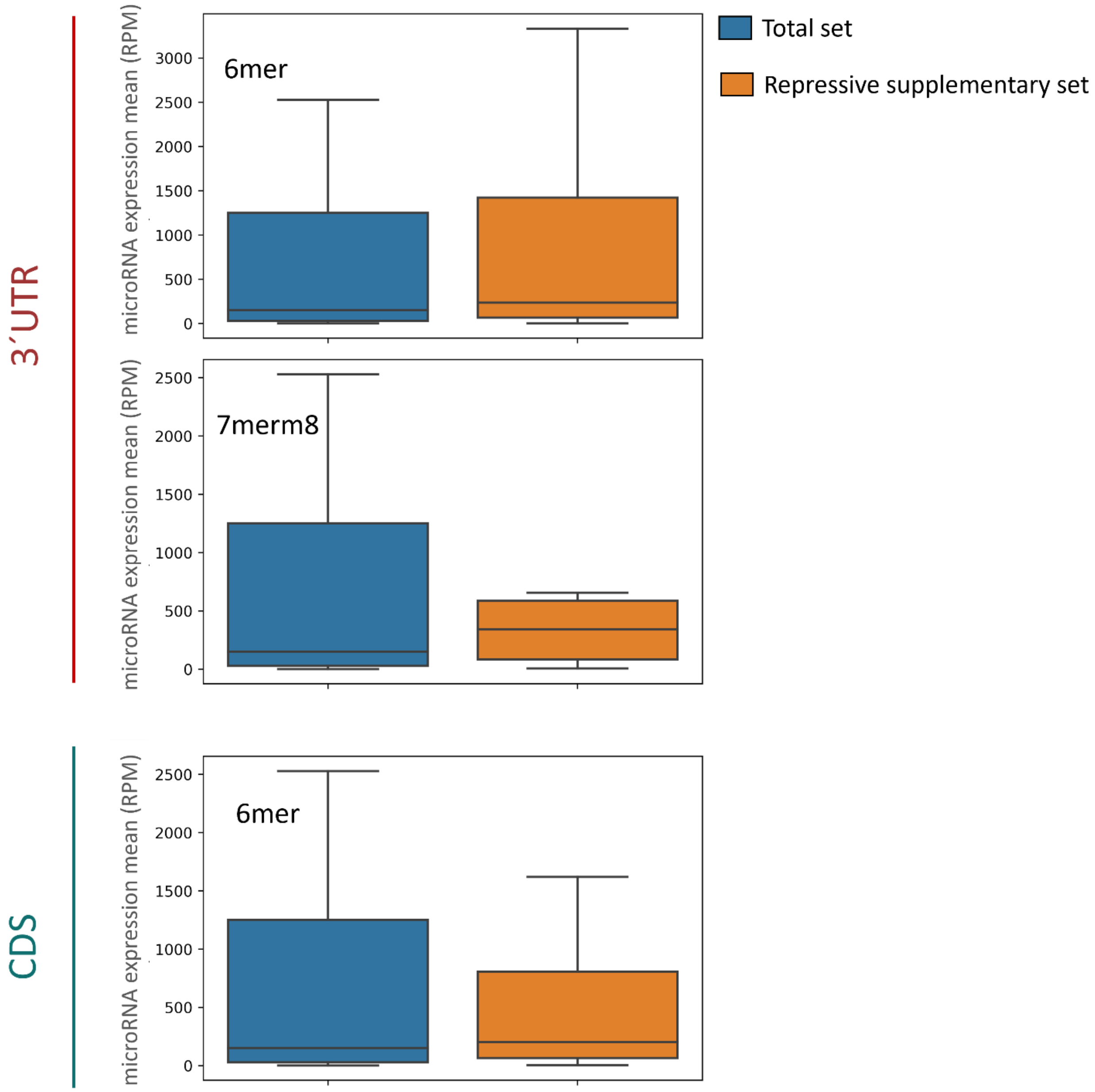

Figure S4. *Mean microRNA expression of the 143 microRNAs studied (blue) and the 39 microRNAs with repressive supplementary interactions (orange).* The interactions at the indicated transcript regions are plotted separately. Only the regions that showed a statistically significant contribution of the 3´-supplementary interactions were included in the analysis (19: 6mer at the 3´UTR, 16: 7mer-m8 at the 3´UTR and 21: 6mer at the CDS).

Trinidad et al., 2022. ***Transcriptome-wide analysis of microRNA-mRNA correlations in unperturbed tissue transcriptomes identifies microRNA targeting determinants.***

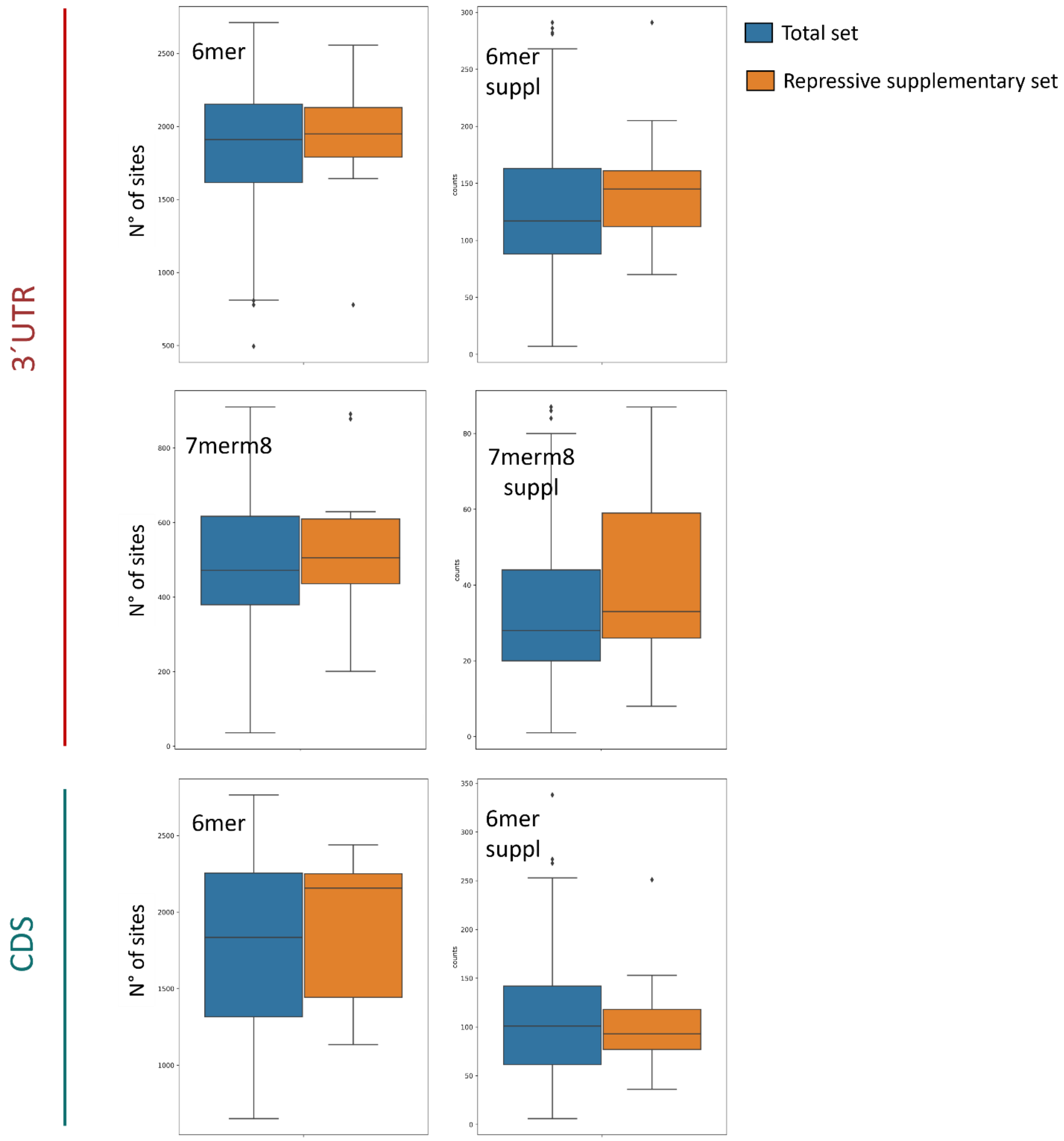

Figure S5. *Number of sites for the 143 microRNAs studied (blue) and the 39 microRNAs with repressive supplementary interactions (orange).* The interactions at the indicated transcript regions are plotted separately. Only the regions that showed a statistically significant contribution of the 3´-supplementary interactions were included in the analysis (19: 6mer at the 3´UTR, 16: 7mer-m8 at the 3´UTR and 21: 6mer at the CDS).

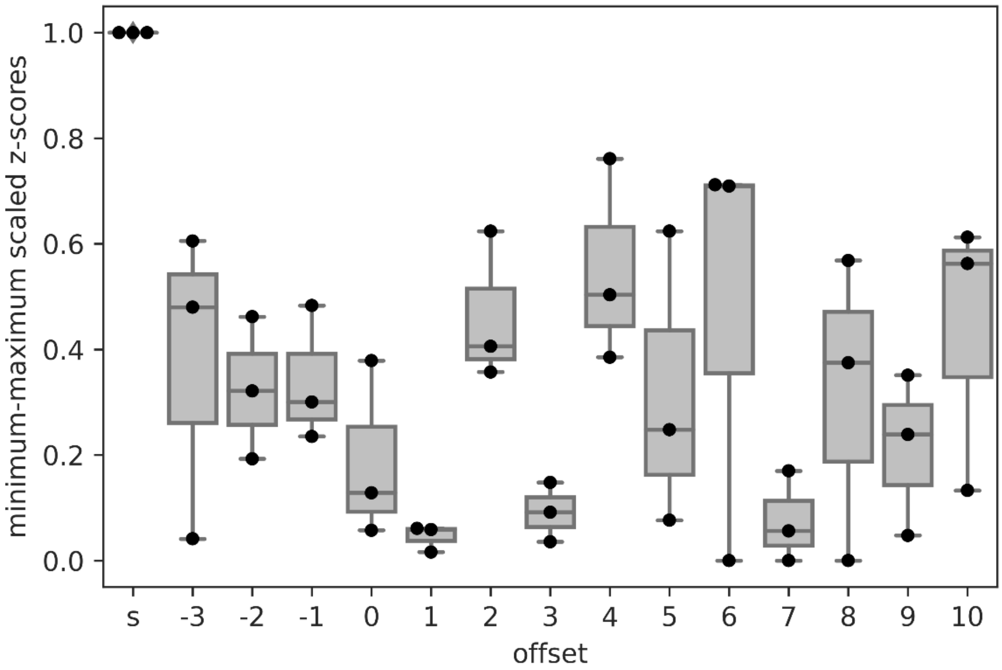

Figure S6. *Distribution of scaled Z-scores by offset of the microRNA/mRNA interaction using 3'-supplementary pairing region.* Each boxplot is composed by 3 datapoints which correspond to the Z-score mean of each interaction (6mer 3'UTR, 7mer-m8 3'UTR, 6mer CDS). Data was minimum-maximum scaled inside each interaction to normalize the different ranges of Z-scores and then we combine the three median of the three Z-scores.
